# Carbohydrate and Energy Composition of Preterm Human Milk: A Systematic Review and Meta-Analysis

**DOI:** 10.1016/j.advnut.2026.100720

**Published:** 2026-08-18

**Authors:** Derek C Miketinas, Mindy A Patterson, Vanessa K Thyne, Dixie L Carter, Nathan A Meredith, Maria Fernanda Nunez, Katie E Niemeier, Jennifer N Kinnaman, Tonya M Bender, Ariana DL Bailey

**Affiliations:** 1 Department of Nutrition and Food Sciences, Texas Woman’s University, Houston, TX, United States; 2 Institute for Women’s Health, Texas Woman’s University, Houston, TX, United States; 3 Global Research & Development, Mead Johnson Nutrition, Evansville, IN, United States

**Keywords:** carbohydrate, lactose, energy, preterm, human milk, milk, colostrum, transition, mature

## Abstract

Preterm human milk (HM) is a major source of carbohydrates (CHOs), primarily lactose, which contribute to energy intake and support growth and neurodevelopment. This systematic review and meta-analyses quantified total CHO, lactose, and energy content in preterm HM. EBSCO (Health Source—Nursing/Academic Edition and MEDLINE with Full Text), PubMed, and Scopus were searched through July 2023 for studies reporting nutrient composition in preterm HM (<37 wk gestation) from countries with a Human Development Index ≥0.8 to reduce potential heterogeneity that may influence HM composition. Cross-sectional, case–control (*n* ≥ 1), prospective cohort, and randomized controlled trials published in English were included. Two reviewers independently screened titles and abstracts. Of the studies meeting inclusion criteria, this meta-analysis included articles that reported total CHO, lactose, and energy content in preterm HM. Random-effects models estimated pooled mean amounts of total CHO, lactose, and energy across studies. Heterogeneity was estimated using *I*^2^ and publication bias using Begg’s rank correlation test. Repeated measures were aggregated. Of 884 identified articles, 53 studies reported total CHO, lactose, and energy. Mean (95% confidence interval) total CHO was 6.74 (6.41, 7.07) g/100 mL with the lowest concentration in colostrum [6.30 (6.13, 6.48) g/100 mL]. Total CHO was similar for transition [6.83 (6.60, 7.06) g/100 mL] and mature [6.80 (6.45, 7.14) g/100 mL] HM. Lactose followed a comparable pattern, with an overall mean of 6.33 (6.02, 6.64) g/100 mL. Mean energy content was 68.13 (65.92, 70.33) kcal/100 mL; colostrum had the lowest, whereas transition and mature were similar. Stratified analyses by analytical method and milk type were also conducted. This systematic review provides updated pooled estimates of total CHO and lactose concentrations and energy in preterm HM and highlights substantial variability related to study design, analytical methods, and data reporting.

This trial was registered at the International Prospective Register of Systematic Reviews (https://www.crd.york.ac.uk/PROSPERO/display_record.php?RecordID=445191) as CRD42023445191.

**Statement of significance**

This article advances the field of preterm human milk (HM) nutrition by providing up-to-date estimates of total carbohydrate, lactose, and energy in colostrum, transition, and mature preterm HM. These values fulfill a critical need for practitioners and researchers to more accurately understand the nutritional needs of preterm infants, as well as any supplementary nutrition that may be necessary for appropriate growth and development.

## Introduction

Energy intake is critical to support growth, neurodevelopment, and vital processes in preterm infants. Because of a shortened gestational period, preterm infants are born with energy and nutrient deficits, resulting in limited glycogen and adipose stores at birth. Human milk (HM) is the recommended base feeding; however, unfortified HM may not provide enough energy to meet growth requirements, prevent growth faltering, or ensure preterm growth trajectory meets term growth by ∼44 wk [1,2]. Preterm infants have also been observed to have abnormal body composition compared with term infants, with excess body fat deposition being associated with excess glucose and lipid provision during the parenteral and enteral feeding periods after birth [3]. Therefore, feeding goals include adequate energy consumption through a balance of macronutrients, including appropriate carbohydrate (CHO) provision to support linear growth and body composition development comparable with expected intrauterine growth patterns.

Of the digestible CHO in preterm and term HM, lactose is the predominant form and represents ∼70%–85% of the total CHO, providing an estimated 30%–40% of the total energy content in HM [4]. Lactose is a necessary nutrient for preterm infants as it supports the absorption of minerals such as calcium and magnesium [3,5]. Previous systematic reviews have reported that the lactose and CHO content of preterm HM slightly but steadily increases over the first ∼4–6 wk before largely stabilizing. Similarly, the energy density of preterm HM has been reported to slightly increase over the course of lactation corresponding with increases in both lipid and CHO content [6].

The primary objective of this systematic review was to estimate the total CHO, lactose, and energy content and variability of preterm HM using a series of meta-analyses, stratified by milk type, and to provide updated estimates for these nutrients because slight differences from prior systematic reviews [[7], [8], [9]] were expected because of the addition of data from more recent studies, differences in inclusion criteria, as well as differences in analytical and statistical methods. The secondary objective was to stratify total CHO, lactose, and energy by analytical method. The broader research aim was to conduct a systematic review and a series of meta-analyses to estimate preterm HM nutrient composition and characteristics.

## Methods

This is the third among a series of meta-analyses that utilized one systematic review process to identify inclusion criteria to estimate the nutrient composition of preterm HM. These meta-analyses examine total CHO, lactose, and energy composition of HM and share similar methodology to the first publication in this series [10]. The protocol was registered at PROSPERO (CRD42023445191; https://www.crd.york.ac.uk/PROSPERO/display_record.php?RecordID=445191).

### Search strategy and eligibility criteria

The search strategy and eligibility criteria were previously published [10]. EBSCO (Health Source—Nursing/Academic Edition and MEDLINE with Full Text), PubMed, and Scopus were searched for articles that reported preterm HM composition. EBSCO and PubMed search terms included ("Milk, Human/chemistry"[Mesh]) AND ("Premature Birth"[MeSH]) NOT ("systematic review"[Publication Type]) AND “Premature Birth” [MeSH Terms] AND "milk, human"[MeSH Terms] OR "Colostrum"[MeSH Terms]. Scopus search terms included TITLE-ABS-KEY ("breast Milk", AND human) AND TITLE-ABS-KEY (preterm OR premature) AND TITLE-ABSKEY ("Breast Milk" AND analysis) AND LIMIT-TO (SUBJAREA, "CHEM"). Gray literature was not included in the search and selection process. The eligibility criteria consisted of studies published in English until 2023 and that reported preterm [defined as gestational age (GA) <37 wk or stated as preterm by authors] HM nutrient composition. Countries [defined as a Human Development Index (HDI) < 0.8] at the time of publication were excluded, donor milk samples were excluded, as well as those studies with *n* = 1. The rationale for excluding studies with an HDI of <0.8 was to reduce heterogeneity that could arise from differences in socioeconomic, nutritional, healthcare, and environmental factors that may influence HM composition. A similar approach was followed by Gidrewicz and Fenton [8], which also excluded studies conducted in developing countries with an attempt to exclude mothers with suboptimal nutritional status.

### Data extraction

Covidence software was used to identify articles meeting inclusion criteria. After duplicates were removed by the software, 2 independent reviewers (DCM and MAP) systematically reviewed the searched titles, abstracts, and full-text articles. Any disagreements were resolved with a third researcher (ADLB).

Three researchers (D.C.M., V.K.T., and M.A.P.) independently extracted the following data from the included studies: name of first author, publication year, country, study design, sample size of infants, GA, age at which HM was collected, milk type (colostrum, transition, and mature but only if specified), HM collection method, and total CHO, lactose, and energy analysis method. Any discrepancies in data extraction were reviewed by reviewers and corrected.

### Statistical analysis

These analyses were restricted to total CHO, lactose, and energy composition. Preterm HM samples were treated as the sampling unit unless not provided; otherwise, the number of infants was used. For studies that reported total CHO, lactose, and/or energy across subgroups, the total sample mean and SD were estimated. Supplemental Table 1 provides the data handling steps for how missing data were treated. SD was imputed for 2 studies (7 entries) for CHO, 2 studies (8 entries) for lactose, and for 2 studies (18 entries) for energy. For randomized controlled trials, baseline or control group milk composition estimates were extracted. Units were converted to g/100 mL for total CHO and lactose and kcal/100 mL for energy. Milk density was assumed to be 1.03 g/mL where necessary, consistent with our previous work [10].

These meta-analyses were conducted in R using the metafor package [11] to fit random-effects models to estimate total CHO, lactose, and energy content across HM type [colostrum (<5 d), transition (5–14 d), and mature (≥14 d)]. Pooled estimates along with 95% confidence intervals (CIs) and 95% prediction intervals (PIs) were calculated. For studies contributing multiple observations (e.g., measurements at multiple time points from the same mothers), estimates were aggregated to the study level using an inverse-variance weighted mean [12,13]. A within-study correlation correction (ρ = 0.6) was applied to the aggregated variance to account for the nonindependence of repeated measures from the same participants, following the approach described by Borenstein et al. [14]. Heterogeneity was tested using Cochrane-*I*^2^ statistics. The level of heterogeneity (*I*^2^) was measured as a percentage where <40% is low, 40%–75% is moderate, and ≥75% is high heterogeneity [15]. Publication bias was assessed using Begg’s rank correlation test (Kendall’s τ) and an examination of the funnel plots. Sensitivity analyses were conducted to evaluate the impact of each study on the pooled estimates. Mixed-effects meta-regression models were fit to explore sources of between-study heterogeneity identified in the primary meta-analyses. Milk type (colostrum, transitional, mature), analytical methodology, and GA at delivery were included as fixed-effect moderators. Models were fit using restricted maximum likelihood in the metafor package in R [11]. The omnibus test statistic (QM) was used to evaluate the joint significance of all moderators, and pseudo-*R*^2^ was calculated as the proportion of between-study variance explained by the model relative to an intercept-only model [16]. Residual heterogeneity was assessed using the QE statistic.

Aggregating studies that utilize differing methods to measure HM composition can introduce analytical bias. Therefore, we extracted the reported analytical methods for CHO, lactose, and energy. Studies reported HM CHO content as either total CHO or lactose and were divided accordingly for analysis. For those studies reporting total CHO, subgroup analysis was conducted based on analytical methodology: infrared spectroscopy, calculation, or other/unspecified. Similarly, studies reporting lactose were further analyzed by those utilizing enzymatic, chromatographic (HPLC, GC-MS, liquid chromatography tandem mass spectrometry, or other/unspecific analytical methods). Energy was either directly measured by bomb calorimetry or indirectly by calculation from values recorded for macronutrients. Subgroup analysis was conducted based on these approaches.

For studies where the reported nutrient was ambiguous (total CHO or lactose), the analytical methodology was used to assign the nutrient measured. For example, all studies using infrared spectroscopy were assumed to measure total CHO even if reported as lactose because this technique cannot distinguish between lactose and HM oligosaccharides (HMOs) [[17], [18], [19]]. Supplemental Table 2 details the studies whose reported nutrient was changed based on analytical methodology, and Supplemental Table 3 includes sensitivity analyses to compare how estimates changed when reclassified compared with author-reported.

## Results

### Study selection

Of the 884 studies identified, 68 were removed as duplicates, 487 were considered irrelevant based on title and abstract review, leaving 329 studies that involved full-text review (Figure 1). Of those, 260 were excluded because of not reporting total CHO, lactose, and/or energy. An additional 15 studies reporting total CHO, lactose, and/or energy were further excluded (5 reported values from other sources, 4 had an HDI of <0.8, 3 combined preterm, term, and/or donor HM, 1 provided results in a figure without values, 1 used fortified HM, and 1 was a case study). Thus, 53 articles were included in the analysis (4 total CHO only, 7 lactose only, 12 energy only, 20 CHO + energy, 9 lactose + energy, and 1 reported CHO + lactose + energy).

**FIGURE 1:**
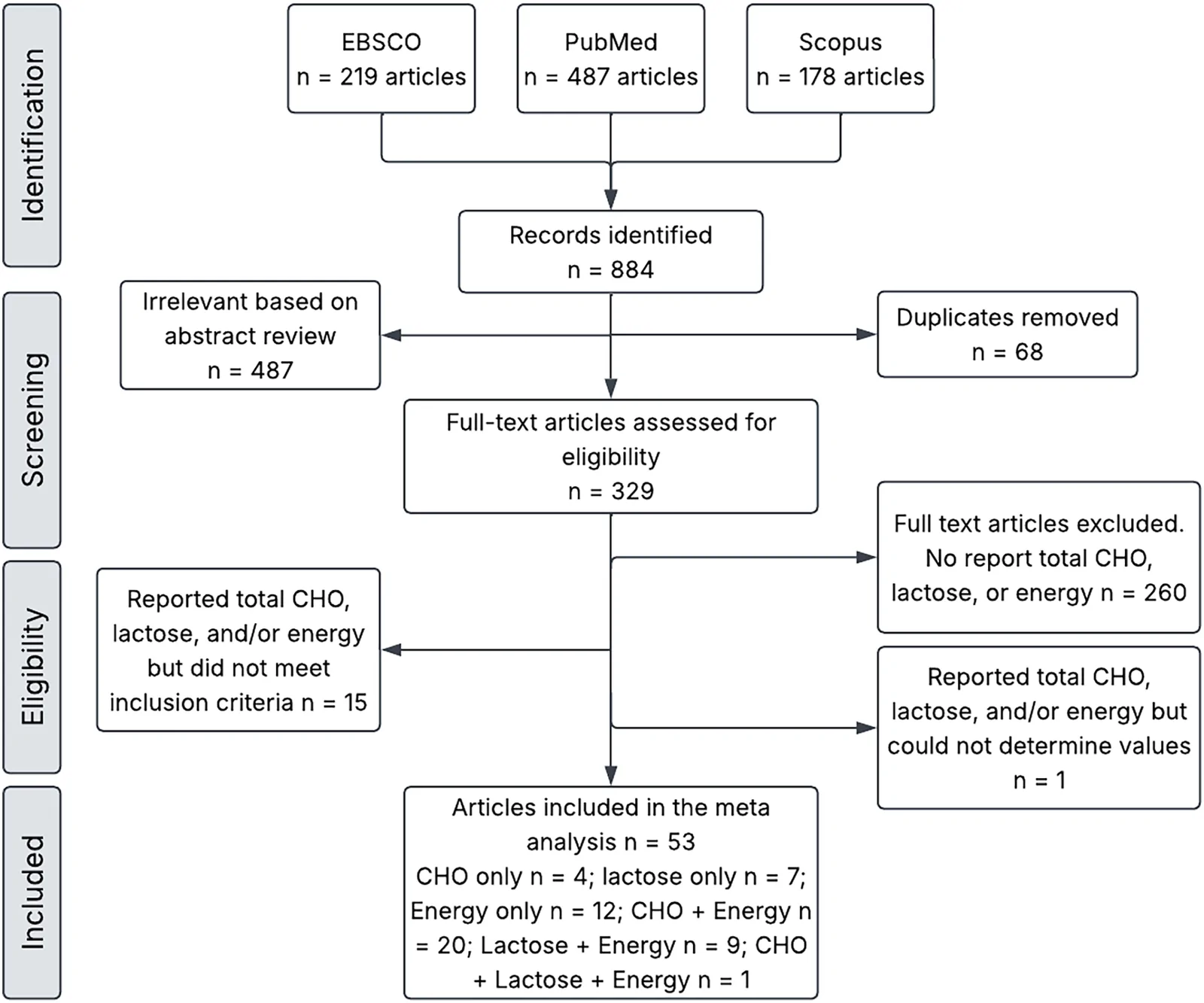
Study selection flowchart.

### Total CHO and lactose

Of the 53 articles, 25 reported total CHO, and 17 reported lactose, with 30 articles providing 2 or more values. Most articles provided 1 or more values for colostrum (<5 d), transition (5–14 d), and mature (≥14 d) HM (Table 1), whereas others did not report or specify the timing of collection. Thus, the number of studies that contributed to each collection phase exceeded the number of articles. The number of studies reporting colostrum, transition, and mature HM for total CHO were 2, 13, and 23, and for lactose were 5, 10, and 14, respectively, and the infant GA range was 23.0–36.0 wk with HM collections across 3–221 d.

**TABLE 1:** Study characteristics total reporting carbohydrate (CHO) and/or lactose in preterm HM (*n* = 40)^a^

Author, First	Year	Country	Study design	Mothers (*n*)	Infants (*n*)	GA^b^ (wk)	HM age^c^ (d)	HM type^d^	Nutrient	Analysis method
Abdulrazzaq, Y [20]	2003	UAE	CS	55	49	<37.0	21	M	CHO^d^	Infrared
Anderson, D [21]	1983	United States	L	14	14	31	(3, 14)	C, T, M	Lactose^e^	Enzymatic
Anderssen, S [22]	2015	Norway	L	28	47	(26.0, 36.0)∗	(13, 30)	T, M	CHO^d^	Infrared
Bauer, J [23]	2011	Germany	L	102	102	28.4	31	M	CHO	Other
Belfort, M [24]	2020	United States	L	34	37	28	58.5	M	CHO	Infrared
Bertino, E [25]	2008	Italy	CS	NR	10	(23.0, 36.0)∗	NR	NR	Lactose	HPLC
Brion, L [26]	2020	United States	RCT	58	58	28	NR	NR	CHO	Infrared
Bulut, O [27]	2019	Turkey	L	32	32	(25.0, 33.0)∗	(5, 39)	C, T, M	CHO^d^	Infrared
Caldeo, V [28]	2021	Ireland	L	29	39	33.7	38.5	M	CHO	Infrared
Campbell-Yeo, M [29]	2010	Canada	RCT	24	24	26.8	31	M	CHO	Calculation
Cavalli, C [30]	2006	Italy	L	17	17	(29.0, 35.6)	(3,29)	M	Lactose	HPLC
Coppa, G [31]	1997	Italy	L	26	26	(27.0, 35.0)	(4, 30)	C, T, M	Lactose	HPLC
de Halleux, V. [32]	2013	Belgium	L	NR	28	NR	28	M	CHO	Infrared
S. de Oliveira, [33]	2017	France	RCT	12	12	30	27	M	CHO	Not specified
Gates, A [6]	2021	United States	L	38	38	28.2	(7, 28)	T, M	CHO	Calculation
Groh-Wargo, S [34]	2016	United States	CS	10	10	(23.6, 32.3)	28∗	M	CHO^d^	Infrared
Gross, S [35]	1980	United States	L	33	NR	31.4	(3, 28)	C, T, M	Lactose	Enzymatic
Gross, S [36]	1983	United States	RCT	NR	20	30.6	(7, 56)	T, M	CHO^d^	Calculation
Gross, S [37]	1987	United States	RCT	10	10	30.3	(7, 21)	T, M	CHO^d^	Calculation
Hsu, Y [38]	2014	Taiwan	L	17	17	29.9	(6, 41)	T, M	CHO^d^	Infrared
Kreissl, A [39]	2016	Austria	L	76	76	(23.2, 31.3)∗	(7, 28)	T, M	CHO^d^	Infrared
Lemons, J [40]	1982	United States	L	20	20	33	(7, 56)	T, M	Lactose	GC mass spec
Lev, H [41]	2014	Israel	L	20	20	30.6	31.5	M	CHO^d^	Infrared
Maas, Y [42]	1998	Netherlands	L	79	79	(25.0, 30.0)	(9, 221)	T, M	CHO	Calculation
Maas, Y [42]	1998	Netherlands	L	79	79	(25.0, 30.0)	(9, 221)	T, M	Lactose	Enzymatic
Maly, J [43]	2019	Czech Republic	L	225	225	(24.0, 35.0)∗	(4, 7)	T	CHO	Infrared
McLeod, G [44]	2013	Australia	L	63	63	(24.0, 27.0)∗	14	T	Lactose	Enzymatic
McLeod, G [45]	2015	Australia	L	27	27	(25.0, 32.0)∗	40	M	Lactose	Enzymatic
Moles, L [46]	2015	Spain	CS	22	22	(24.0, 27.0)	(4, 35)	C, M	Lactose	GC mass spec
Moran-Lev, H [47]	2015	Israel	L	32	32	30.1	31.5	M	CHO	Infrared
Morton, J [48]	2012	United States	L	67	52	<31.0	(7, 56)	T, M	Lactose	Enzymatic
Perrella, S [49]	2015	Australia	RCT	25	23	30.1			Lactose^e^	Enzymatic
Radmacher, P [50]	2013	United States	CS	NR	83	NR	(7, 35)	T, M	CHO^d^	Infrared
Saarela, T [51]	2005	Finland	L	36	36	31.4	(7, 180)	T, M	Lactose	Enzymatic
Sahin, S [52]	2020	Turkey	L	39	39	29.7	(3, 28)	C, T, M	CHO	Infrared
Sann, L [53]	1981	France	L	41	41	(26.0, 35.0)∗	(4, 15)	C, T, M	Lactose	HPLC
Sauer, C [54]	2017	United States	L	18	24	29	31	M	Lactose^e^	LC-MS/MS
Smilowitz, J [55]	2014	United States	L	5	NR	(26.0, 36.0)	185	M	CHO^d^	Infrared
Stein, H [56]	1986	South Africa	RCT	11	11	33.5	(5, 33)	T, M	Lactose	Not specified
Stolz Sjöström, E [57]	2014	Sweden	L	256	256	25.3	(6, 106)	T, M	CHO	Infrared
Thomas, M [58]	1986	United States	L	20	NR	(30.0, 34.0)	16	M	Lactose	Enzymatic
Zachariassen, G [59]	2013	Denmark	L	214	214	(<32.0)	(14, 18)	T, M	CHO^d^	Infrared

Abbreviations: C, colostrum; CS, cross-sectional; GA, gestational age; HM, human milk; L, longitudinal; LC-MS/MS, liquid chromatography tandem mass spectrometry; M, mature; NR, not reported; RCT, randomized controlled trial; T, transitional; UAE, United Arab Emirates.

a Values reported as mean or median∗ or (range) or (min, max) or (IQR range)∗.

b Mean gestational age. Parentheses indicate upper and lower ranges.

c Postpartum day HM was collected.

d Authors originally reported lactose but this was changed to total carbohydrate given analytical method reported.

e Authors originally reported total carbohydrate but this was changed to total carbohydrate given analytical method reported.

Figure 2 represents the forest plots for total CHO content (g/100 mL) in preterm HM across milk type (Panel A = colostrum; Panel B = transition; Panel C = mature). The meta-analysis showed the pooled mean for total CHO concentration of preterm HM samples as: 6.74 (95% CI: 6.41, 7.07; 95% PI: 5.12, 8.35) g/100 mL with a high heterogeneity among studies (*I*^2^ = 99.8%). When stratified by milk type, the estimates for colostrum, transition, and mature HM CHO were 6.30 (6.13, 6.48) g/100 mL (*I*^2^ = 0.0%), 6.83 (6.60, 7.06) g/100 mL (*I*^2^ = 97.9%), and 6.80 (6.45, 7.14) g/100 mL (*I*^2^ = 99.9%), respectively. The high heterogeneity among the studies reflected lactation stage at HM collection (range 3 d to 31.6 wk), feeding phase (foremilk, hindmilk, or pooled), storage method (room temperature, 4°C, −70°C), and duration of storage (range 1–4 d at 4°C).

**FIGURE 2:**
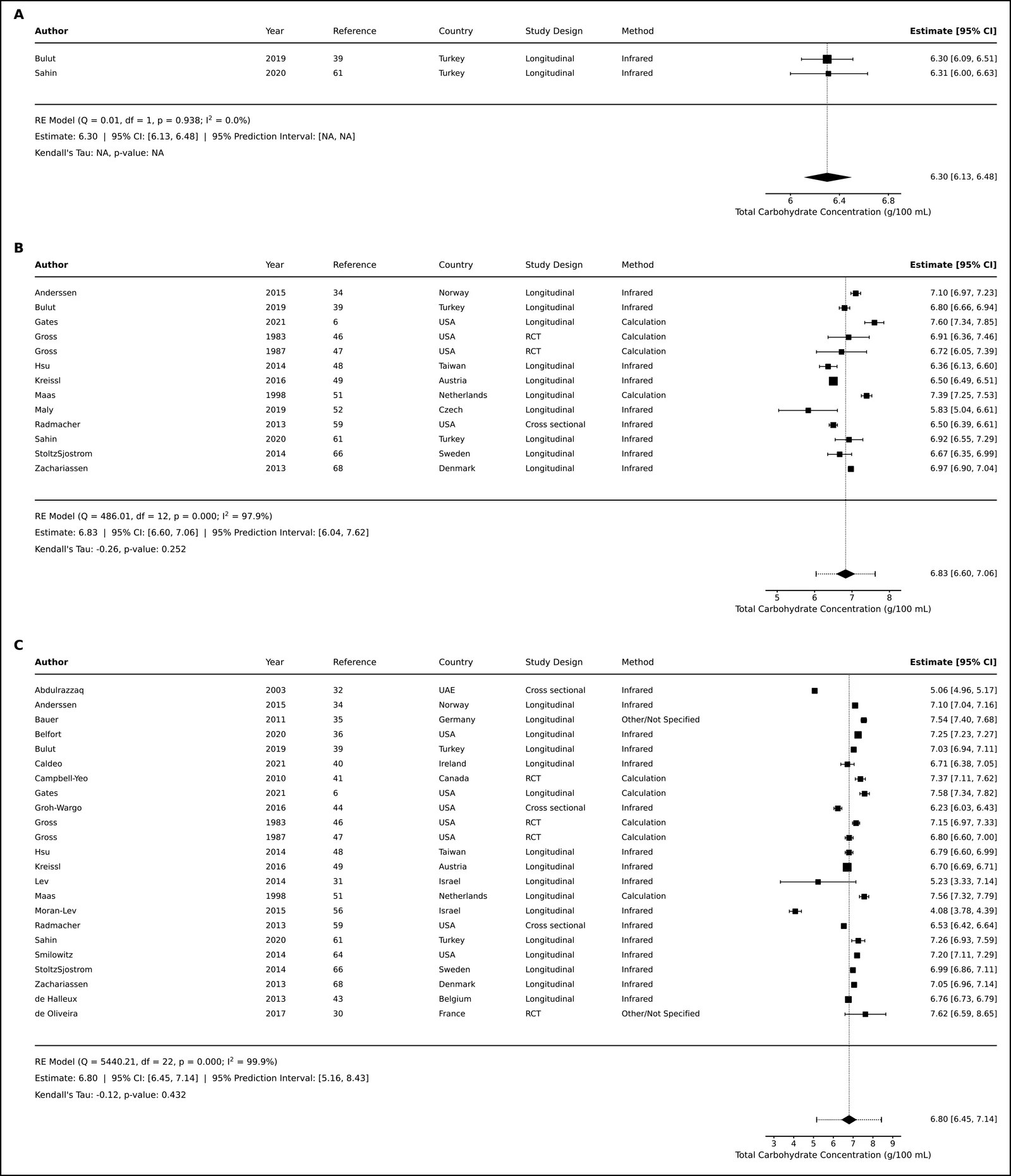
Forest plots for total carbohydrate content (g/100 mL) in preterm human milk (HM) across milk type. (A) Colostrum HM (<4 d), RE Model (Q = 0.01, df = 1, *P* = 0.0938; *I*^2^ = 0.0%); Kendall’s τ: 1.000, *P* = 1.00. (B) Transition HM (5–14 d), RE Model (Q = 486.01, df = 12, *P* = 0.000, *I*^2^ = 97.9%); Kendall’s τ: −0.26, *P* = 0.252. (C) Mature HM (≥14 d), RE Model (Q = 5440.21, df = 22, *P* = 0.000, *I*^2^ = 99.9%); Kendall’s τ: −0.12, *P* = 0.432.

Figure 3 provides the forest plots for total lactose [6.33 (95% CI: 6.02, 6.64; 95% PI: 5.11, 7.55) g/100 mL, *I*^2^ = 96.1%] concentrations in preterm HM across time. Panel A, colostrum, reports the lowest lactose [5.78 (5.43, 6.12) g/100 mL, *I*^2^ = 63.5%], with similar amounts for transition [Panel B, 6.63 (6.32, 6.94) g/100 mL, *I*^2^ = 93.7%] and mature [Panel C, 6.57 (6.26, 6.89) g/100 mL, *I*^2^ = 94.0%] HM. Sensitivity analyses did not show any noticeable influence of individual studies on CHO or lactose estimates (Supplemental Figure 1). Forest plots by analytical method and milk type are provided in Supplemental Figure 2.

**FIGURE 3:**
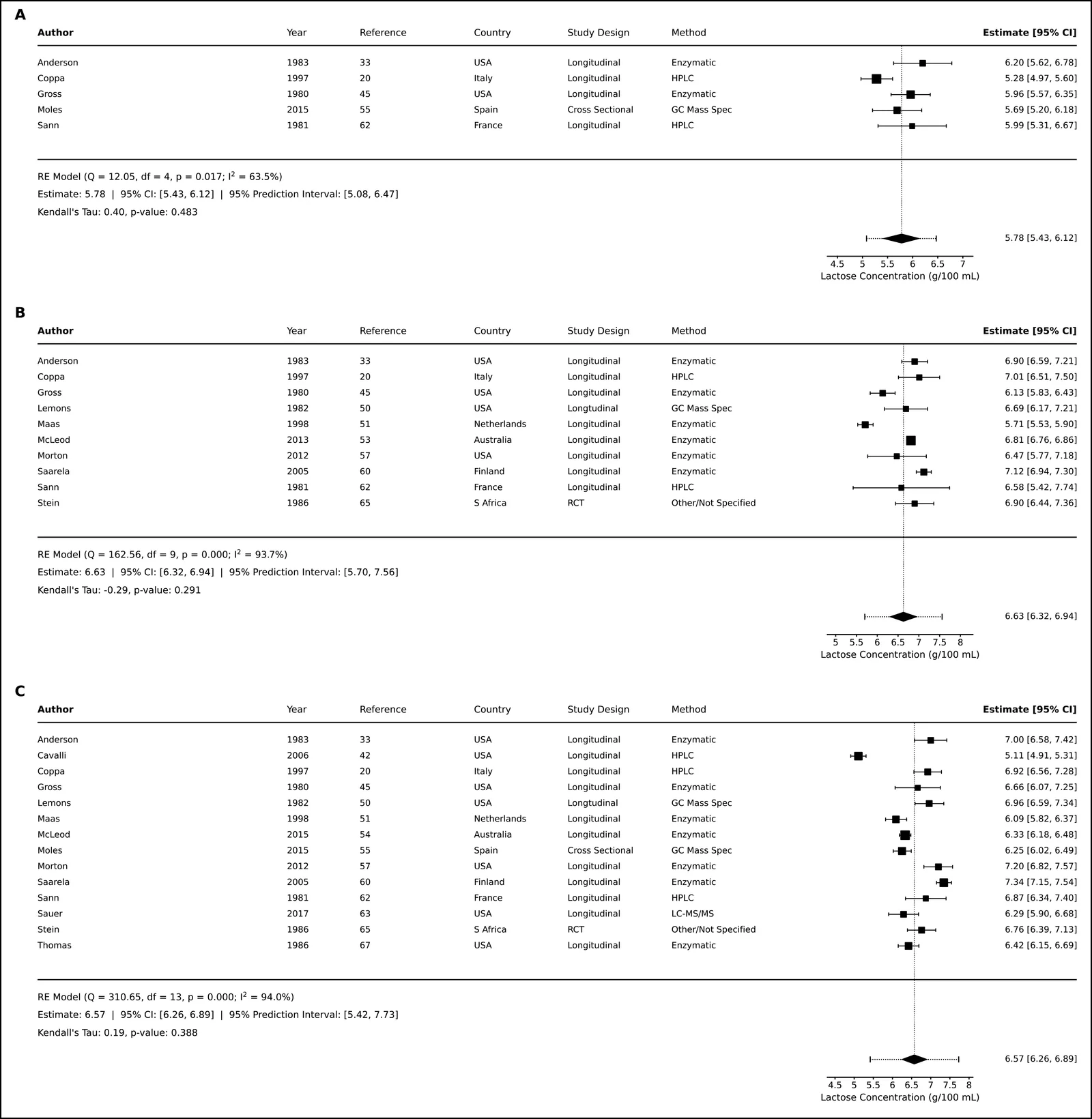
Forest plots for total lactose content (g/100 mL) in preterm human milk (HM) across milk type. (A) Colostrum HM (<4 d), RE Model (Q = 12.05, df = 4, *P* = 0.017; *I*^2^= 63.5%); Kendall’s τ: 0.40, *P* = 0.483. (B) Transition HM (5–14 d), RE Model (Q = 162.56, df = 9, *P* = 0.000, *I*^2^ = 93.7%); Kendall’s τ: −0.29, *P* = 0.291. (C) Mature HM (≥14 d), RE Model (Q = 310.65, df = 13, *P* = 0.000, *I*^2^ = 94.0%); Kendall’s τ: 0.19, *P* = 0.388.

### Energy

A summary of study characteristics reporting energy can be found in Table 2. A total of 42 studies reported energy, where many provided ≥1 value stratified by HM type: colostrum (*n* = 9); transition (*n* = 26); mature (*n* = 32). The data collections occurred between 23.2–36.0 wk GA and 0–180 d. Energy composition across all studies was 68.13 (95% CI: 65.92, 70.33; 95% PI: 55.08, 81.17) kcal/100 mL, *I*^2^ = 97.2% (Panel **A**). Figure 4 provides the forest plots for the estimated mean energy composition across milk type. Colostrum had the lowest energy [Panel A; 64.38 (57.89, 70.87 kcal/100 mL, *I*^2^ = 97.16%], whereas transition [Panel B; 69.47 (66.19, 72.74) kcal/100 mL, *I*^2^ = 97.48%] and mature [Panel C; 68.70 (66.13, 71.28) kcal/100 mL, *I*^2^ = 98.7%] had similar energy content. Sensitivity analyses did not show any noticeable influence of individual studies’ energy density estimates (Supplemental Figure 1). However, there was some evidence of publication bias for all studies (τ = 0.28, *P* = 0.010), and evidence of asymmetry was observed in the funnel plot (Supplemental Figure 3).

**TABLE 2:** Study characteristics reporting energy in preterm HM (*n* = 43)^a^

Author, First	Year	Country	Study design	Mothers (*n*)	Infants (*n*)	GA^b^ (wk)	HM age^c^ (d)	HM type^d^	Analysis method
Anderson, D [21]	1983	United States	L	14	14	31	(3, 14)	C, M	Bomb calorimetry
Bauer, J [23]	2011	Germany	L	102	102	28.4	6	T	Bomb calorimetry
Belfort, M [24]	2020	United States	L	34	37	28	58.5	M	Calculated
Bishara, R [60]	2008	United States	L	24	30	30	24	M	Bomb calorimetry
Brion, L [26]	2020	United States	RCT	58	58	28.3	NR	NR	Calculated
Bulut, O [27]	2019	Turkey	L	32	32	(25.0, 33.0)	(7, 42)	T, M	Calculated
Butte, N [61]	1984	United States	L	8	8	33.9	(14, 84)	T, M	Bomb calorimetry
Caldeo, V [28]	2021	Ireland	L	29	39	33.5	38.5	M	Calculated
Campbell-Yeo, M [29]	2010	Canada	RCT	24	24	26.8	(0, 14)	C, T	Not specified
de Halleux, V. [32]	2013	Belgium	L	NR	28	28.6	28	M	Calculated
Faerk, J [62]	2001	Denmark	L	101	101	28	(11, 67)	T, M	Calculated
Gates, A [6]	2021	United States	L	38	38	28.2	(7, 28)	T, M	Bomb calorimetry
Groh-Wargo, S [34]	2016	United States	CS	NR	10	(23.6, 32.3)	28∗	M	Calculated
Gross, S [35]	1980	United States	L	33	NR	31.4	(3, 28)	C, T, M	Calculated
Gross, S [36]	1983	United States	RCT	NR	20	30.6	(7, 81)	T, M	Calculated
Gross, S [37]	1987	United States	RCT	10	10	30.3	(7, 21)	T, M	Calculated
Hsu, Y [38]	2014	Taiwan	L	17	17	29.9	(4, 25)	C, M	Calculated
Kociszewska-Najman, B [63]	2012	Poland	L	22	22	(26.0, 32.0)	(3, 15)	C, T, M	Calculated
Kreissl, A [39]	2016	Austria	L	76	76	(23.2, 31.3)∗	(7, 28)	T,M	Calculated
Lemons, J [40]	1982	United States	L	20	20	33	(7, 56)	T, M	Bomb calorimetry
Lepage, G [64]	1984	Canada	CS	32	32	(26.0, 36.0)	(5, 30)	M	Bomb calorimetry
Lev, H [41]	2014	Israel	L	20	20	30.6	31.5	M	Calculated
Lin, H [65]	2011	Taiwan	L	14	14	(27.0, 36.0)∗	15	M	Bomb calorimetry
Maas, C [66]	2017	Germany	RCT	60	60	(<32.0)∗	(14, 28)	T, M	Calculated
Maas, Y [42]	1998	Netherlands	L	79	79	(25.0, 29.9)	(9, 53)	T, M	Calculated
Maly, J [42]	2019	Czech Republic	L	225	225	(24.0, 27.0)∗	(4,7)	T	Calculated
McLeod, G [44]	2013	Australia	L	63	63	(24.0, 27.0)∗	14	T	Calculated
McLeod, G [45]	2015	Australia	L	27	27	(25.0, 32.0)∗	40	M	Calculated
Meier, P [67]	2002	United States	L	17	17	28.4	19.9	M	Bomb calorimetry
Moran-Lev, H [47]	2015	Israel	L	32	32	30.1	31.5	M	Calculated
Morton, J [48]	2012	United States	L	67	52	(<31.0)	(4, 53)	C, T, M	Calculated
Norrgrann, M [68]	2023	Sweden	CS	12	12	28.1∗	9.5	T	Calculated
Perrella, S [49]	2015	Australia	RCT	25	23	30.1	NR	NR	Calculated
Radmacher, P [50]	2013	United States	CS	NR	83	NR	(7, 35)	T, M	Calculated
Saarela, T [51]	2005	Finland	L	36	36	31.4	(7, 180)	T, M	Calculated
Sahin, S [52]	2020	Turkey	L	39	39	29.7	(3, 28)	C, T, M	Calculated
Sauer, C [54]	2017	United States	L	18	18	31	11	T	Calculated
Silber, G [69]	1988	United States	L	5	5	29.6	(0, 4)	C	Bomb calorimetry
Smilowitz, J [55]	2014	United States	L	5	NR	(26.0, 36.0)	185	M	Calculated
Stein, H [56]	1986	South Africa	RCT	11	11	NR	(5, 33)	T, M	Not Specified
Stoltz Sjöström, E. [57]	2014	Sweden	L	256	256	(<27.0)	(6, 106)	T, M	Calculated
Whyte, R [70]	1983	Canada	L	NR	9	30	(1, 14)	C, T	Bomb calorimetry
Zachariassen, G [59]	2013	Denmark	L	214	214	(<32.0)	(14, 84)	T, M	Calculated

Abbreviations: C, colostrum; CS, cross-sectional; GA, gestational age; HM, human milk; L, longitudinal; M, mature; NR, not reported, RCT, randomized controlled trial; T, transitional.

a Values reported as mean or (min, max) or (IQR range)∗.

b Mean gestational age. Parentheses indicate upper and lower ranges.

c Postpartum day HM was collected.

d Listed only if article specified.

**FIGURE 4:**
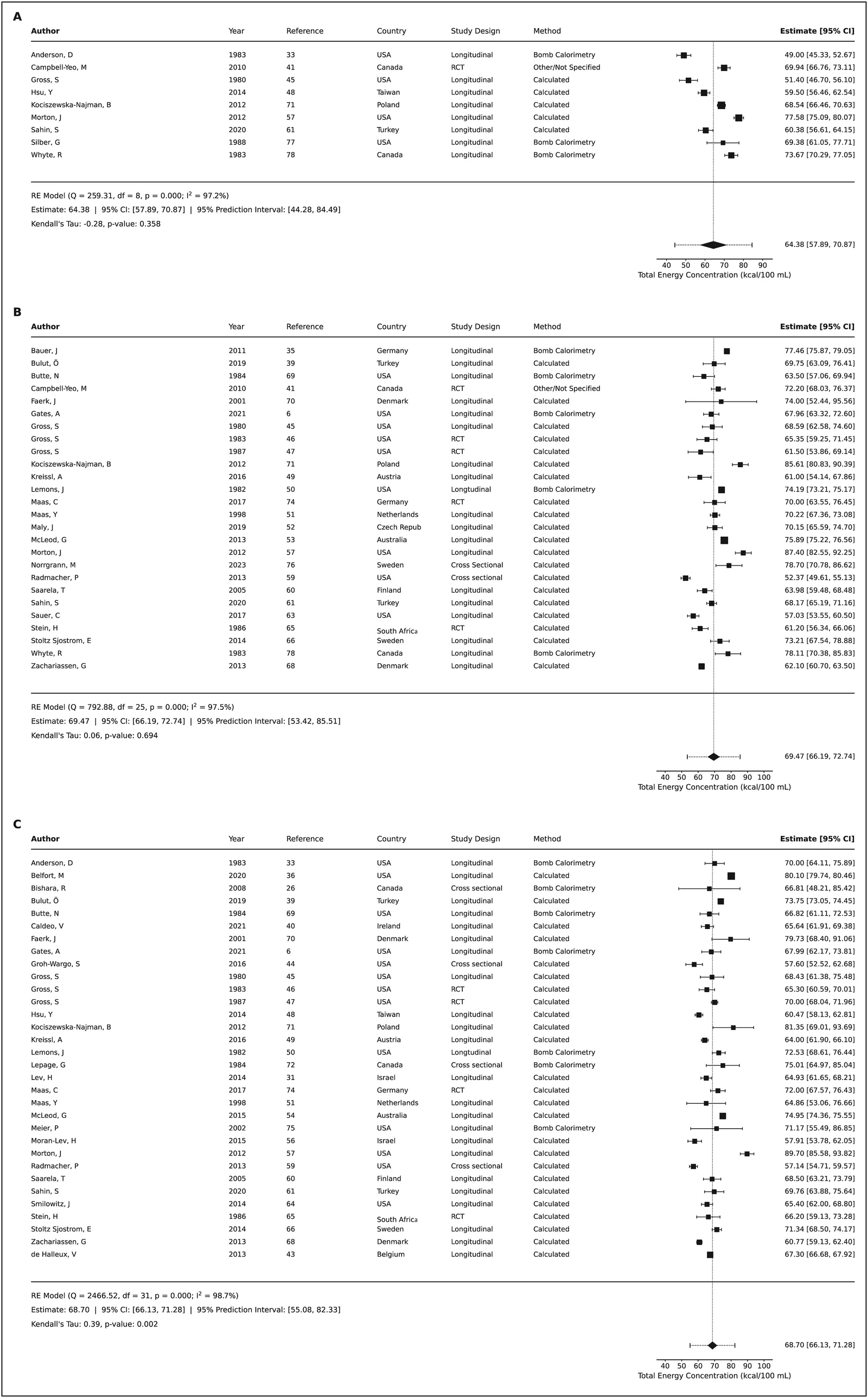
Forest plots for total energy content (g/100 mL) in preterm human milk (HM) across milk type. (A) Colostrum HM (<4 d), RE Model (Q = 259.31, df = 8, *P* = 0.000; *I*^2^ = 97.16%); Kendall’s τ: −0.28, *P* = 0.0.358. (B) Transition HM (5–14 d), RE Model (Q = 792.88, df = 25, *P* = 0.000, *I*^2^ = 97.5%); Kendall’s τ: 0.06, *P* = 0.694. (C) Mature HM (≥14 d), RE Model (Q = 2466.52, df = 31, *P* = 0.000, *I*^2^ = 98.66%); Kendall’s τ: 0.387, *P* = 0.002.

### Total CHO content by analytical methodology

Analysis of total CHO by infrared spectroscopy was the most common technique across studies (*n* = 18) followed by indirect calculation (*n* = 5) and other/nonspecified (*n* = 2). Overall/not specified included a study by Bauer and Gerss [23] that performed orcinol colorimetric analysis to quantify total CHO and another by de Oliveira et al. [33] that did not provide an analytical method for total CHO. Overall, the other/nonspecified methods provided the highest total mean HM CHO value at 7.54 (7.40, 7.68) g/100 mL (*I*^2^ = 0%), followed by indirect calculation at 7.32 (7.05, 7.58) (*I*^2^ = 80.1%), and then infrared, which provided the lowest at 6.50 (6.10, 6.90) g/100 mL (*I*^2^ = 99.8%). Total CHO content by analytical method and milk type (C, T, M) can be found in Supplemental Table 4 with corresponding forest plots in Supplemental Figure 2.

### Lactose content by analytical methodology

Traditional enzymatic methods were most prevalent for measurement of lactose specifically, although newer chromatographic technologies, which offer the ability to measure a full monosaccharide and disaccharide profile, are starting to gain acceptance and see more frequent use by the HM analytical community. The method producing the highest lactose value was other/not specified [6.90 (6.48, 7.31) g/100 mL, *I*^2^ = 21.7%]. Enzymatic and GC-MS resulted in similar lactose values [6.48 (6.09, 6.87) g/100 mL, *I*^2^ = 96.8% and 6.46 (5.56, 7.36) g/100 mL, *I*^2^ = 83.4%, respectively]. The lowest lactose concentration of 5.79 (5.01, 6.39) g/100 mL (*I*^2^ = 81.6%) resulted from HPLC analysis. Lactose by analytical method and milk type (C, T, M) can be found in Supplemental Table 4 with corresponding forest plots in Supplemental Figure 2.

### Total energy content by analytical methodology

Determination of total energy by calculation from the concentration of macronutrients was significantly more common (*n* = 31) than direct measurement by bomb calorimetry (*n* = 10) or other methods (*n* = 1). Calculating energy provided the lowest energy [67.31 (64.65, 69.97) kcal/100 mL, *I*^2^ = 98.8%] than other/nonspecified method (70.77 kcal/100 mL) and bomb calorimetry [71.62 (68.33, 74.92) kcal/100 mL, *I*^2^ = 70.5%]. Total energy by either calorimetry or calculated across milk type (C, T, M) can be found in Supplemental Table 4 with corresponding forest plots in Supplemental Figure 2.

### Meta-regression

Milk type, analytical method, and GA jointly explained a significant proportion of between-study variance for total CHO (QM = 15.2, *P* = 0.010, pseudo-*R*^2^ = 22.8%) and energy (QM = 15.4, *P* = 0.008, pseudo-*R*^2^ = 15.6%) but not for total lactose (QM = 12.2, *P* = 0.095, pseudo-*R*^2^ = 17.5%) (Table 3). Substantial residual heterogeneity remained unaccounted for after adjustment (all QEs *P* < 0.001).

**TABLE 3:** Meta-regression model coefficients and model fit statistics for total carbohydrate, lactose, and energy^a^

Total Carbohydrate	Term	*β*	SE	Z	*P* value	95% CI
	Intercept	9.13	1.46	6.27	<0.001	[6.28, 11.99]
	Milk type: colostrum (ref)	—	—	—	—	—
	Milk type: transitional	0.22	0.51	0.43	0.668	[−0.78, 1.22]
	Milk type: mature	0.34	0.5	0.67	0.5	[−0.64, 1.31]
	Method: infrared (ref)	—	—	—	—	—
	Method: calculation	0.6	0.27	2.25	0.024	[0.08, 1.13]
	Method: other/not specified	0.89	0.54	1.65	0.1	[−0.17, 1.96]
	Gestational age (wk)	−0.10	0.05	−2.05	0.041	[−0.19, −0.004]
QM Omnibus	QM *P* value	QE residual	QE residual *P* value	Pseudo-*R*^2^	τ^2^ residual	
15.2	0.010	5949.8	<0.001	22.8	0.42	

Total lactose	Term	*β*	SE	Z	*P* value	95% CI

	Intercept	3.65	1.58	2.31	0.021	[0.56, 6.74]
	Milk type: colostrum (ref)	—	—	—	—	—
	Milk type: transitional	0.71	0.32	2.24	0.025	[0.09, 1.34]
	Milk type: mature	0.69	0.3	2.31	0.021	[0.10, 1.27]
	Method: enzymatic (ref)	—	—	—	—	—
	Method: GC mass spec	0.01	0.31	0.03	0.978	[−0.61, 0.62]
	Method: HPLC	−0.36	0.28	−1.30	0.195	[−0.90, 0.18]
	Method: tandem MS	−0.25	0.58	−0.44	0.66	[−1.39, 0.88]
	Method: other/not specified	−0.07	0.45	−0.16	0.873	[−0.95, 0.81]
	Gestational age (wk)	0.08	0.05	1.46	0.145	[−0.03, 0.18]
QM Omnibus	QM *P* value	QE residual	QE residual *P* value	Pseudo-*R*^2^	τ^2^ residual	
12.2	0.095	383.3	<0.001	17.5	0.26	

Energy	Term	*β*	SE	Z	*P* value	95% CI

	Intercept	99.63	11.28	8.83	<0.001	[77.53, 121.74]
	Milk type: colostrum (ref)	—	—	—	—	—
	Milk type: transitional	6.27	2.90	2.16	0.031	[0.58, 11.95]
	Milk type: mature	6.02	2.86	2.10	0.035	[0.41, 11.63]
	Method: bomb calorimetry (ref)	—	—	—	—	—
	Method: calculated	–3.03	2.21	–1.37	0.170	[−7.35, 1.30]
	Method: other/not specified	–1.54	5.75	–0.27	0.789	[−12.80, 9.73]
	Gestational age (wk)	–1.12	0.36	–3.15	0.002	[−1.82, –0.42]
QM Omnibus	QM *P* value	QE residual	QE residual *P* value	Pseudo-*R*^2^	τ^2^ residual	
15.4	0.009	2232.4	<0.001	15.6	49.20	

a Mixed-effects meta-regression models were fit to explore sources of between-study heterogeneity identified in the primary meta-analyses. Milk type (colostrum, transitional, mature), analytical methodology, and gestational age at delivery were included as fixed-effect moderators.

## Discussion

Similar to previous systematic reviews [[7], [8], [9]], the current analysis revealed that the energy density of preterm HM was lowest during the first phase of lactation (colostrum), highest during the transition phase, and remaining fairly stable throughout the mature phase. Our estimates for transition and mature energy density (69.36 and 68.85 kcal/100 mL, respectively) were comparable with those of Boyce et al. [7], who reported a calculated energy mean of 65.6 kcal/100 mL for weeks 2–8 of lactation. However, our estimate for colostrum (66.46 kcal/100 mL) was much higher than their reported value of 57.11 kcal/100 mL and higher than the estimates reported by Mimouni et al. [9] (58.8 kcal/100 mL) and Gidrewicz and Fenton [8] (60 kcal/100 mL for the first week of lactation). Conversely, our energy density estimates for transition and mature milk were lower than the estimates reported by Mimouni et al. [9] (74.53 kcal/100 mL) and lower than the estimates reported by Gidrewicz and Fenton [8] for weeks 2 and 3–4 of lactation (71 and 77 kcal/100 mL, respectively). This may be due to the incorporation of newer studies and/or differences in inclusion criteria and statistical methods.

The 95% PIs for energy, CHO, and lactose were wider than the CIs, which highlights the high variability in these estimates. For example, the pooled energy density for all milk types was 68.14 kcal/100 mL (CI: 65.94, 70.93) and the PI spanned ≥26 kcal/100 mL (55.09–81.20 kcal/100 mL). This emphasizes the need for caution when utilizing these pooled mean estimates as benchmarks for preterm infants. Finally, all asymmetry tests should be interpreted with caution in the presence of high heterogeneity between studies.

Although lactose represents the main available CHO source in HM, small amounts of monosaccharides such as glucose are also present [3], which were not individually captured as part of this analysis. However, in one study, it was estimated that monosaccharides made up 1%–2% of total CHO in preterm HM, estimated at 0.60 g/L after 30 d [31]. In addition, the indigestible CHO, HMOs, are a major component of HM, behind only lipids and lactose. Indeed, Coppa et al. [31] noted that HMOs ranged from 25.61 g/L in colostrum and decreased to 16.83 g/L in mature preterm HM, whereas Rozé et al. [71] noted an average concentration of 8523.4 mg/L in preterm HM across the first 7 wk of life. Although HMOs are captured in total CHO because of methodology, an estimate was not determined for HMO components alone in this analysis. Of the published systematic reviews, only one distinguished between lactose and total CHO content [7], whereas the others reported total lactose content as a proxy for total CHO [8,9]. As expected, and like Boyce et al. [7], the estimated total CHO content (6.74 g/100 mL) was higher than lactose (6.37 g/100 mL) in the present analysis with gradual increases across each stage of lactation before stabilizing toward the end of transition and into the mature phase. It is likely that the total CHO measured by infrared methods substantially reflect the presence of HMOs; however, this extrapolation from the present analysis differs from reported HMO amounts, which may be a result of variability in infrared measurements of total CHO or HMO interference with lactose measurements [18,72]. Additionally, our mean estimates for total CHO across lactation were lower than what has been reported by previous systematic reviews. As with energy density, this could be attributed to maternal genetics and health status [73,74] and other factors, including the inability to distinguish lactose from total CHO [[7], [8], [9]], which could have inflated the latter estimate, and/or because of differences in statistical analyses.

The rapid adoption of infrared spectroscopy-based HM analyzers has driven the prevalence of this technique for the analysis of milk macronutrients because of their ease of use and ability to simultaneously measure fat, protein, and total CHO, which may then be used to calculate the total energy content of the sample. This particular methodology has several limitations, including its inability to differentiate between lactose and HMOs [17], which renders it only capable of estimating total CHO despite HMOs accounting for ∼20% of the total CHO in HM [75]. Additionally, the calculation of energy depends on the measured values of the 3 macronutrients, and any measurement bias of these values would inevitably be propagated to energy. Indeed, the present analysis found that the calculation of energy tends to result in a slightly lower value (67.68 kcal/100 mL) compared with direct measurements such as bomb calorimetry (71.62 kcal/100 mL). This finding is similar to what has been previously reported by Gidrewicz and Fenton [8], who reported that bomb calorimetry had higher energy values compared with calculated with differences as small as −2% up to as large as −13% depending on the week of lactation. Furthermore, energy differences can be as high as 20% (3 kcal/oz) based on calculated method [72], showing the importance of standardizing methods and even deciphering between metabolizable energy (recommended) and gross energy. In this study, metabolizable energy was not determined, which can differ by ≤15% of gross energy [76].

### Strengths and limitations

The findings of this meta-analysis and systematic review have several strengths. First, studies from countries with an HDI ≥ 0.8 were included to reduce the potential for heterogeneity that may arise from differences in socioeconomic, nutritional, and environmental differences that can influence HM composition. However, including only studies in very highly developed countries may limit globally generalized results. In addition, the CHO, lactose, and energy values were analyzed by milk type to show differences across lactation, as well as by analytical method to show differences from the method of reference compared with other methods.

Several limitations should be acknowledged. Milk type, collection, and storage all greatly affect nutrient density in HM. In this study, 2 studies [60,72] reported hindmilk being more energy dense than foremilk with large variability as indicated with wide CIs. This is problematic when reporting energy of pooled samples as it does not decipher the differences observed across a single feed. Also, not all studies calculating energy of preterm HM report the macronutrient conversion factors used to calculate energy. For example, the Atwater general factors are 4 kcal/g for CHO and protein and 9 kcal/g for fat [77], whereas the Food and Drug Administration–approved midinfrared HM analyzer uses 4.4 kcal/g protein, 4 kcal/g CHO, and 9.25 kcal/g fat [78]. Another limitation was grouping analytical methods that were not standard or not described into a single “other/not specified” category, which limits interpretability, but we provided them for completeness nonetheless; therefore, these estimates should be viewed as exploratory. Finally, the asymmetry tests should be interpreted with caution given the presence of high heterogeneity between studies.

Substantial differences in CHO and lactose levels are evident with varying storage and processing conditions of HM [33,41]. The highest CHO and lactose levels are reported in fresh HM, whereas significant losses could be seen (∼30%) in frozen milk [41]. Pasteurization also results in lower total CHO levels in comparison with raw HM [33]. These variabilities in storage and processing methods are contributing factors to the large CIs reported in studies, which consequently impacts the estimates captured in the present analysis. Another limitation to note is that some infrared devices are calibrated for bovine milk, which has a very different nutrient matrix compared with HM and can be inaccurate for lactose quantification [17,18]. These devices must therefore be calibrated using HM collected at different lactation phases (foremilk, midmilk, and hindmilk) to provide the most accurate nutrient composition estimates [17]. It is unclear whether each study included in this review that used infrared analysis undertook the proper calibration steps necessary before analyzing the HM samples. More work is needed by the broader HM research community to emphasize the importance and necessity of a standardized analytical approach in future studies.

## Author contributions

The authors’ responsibilities were as follows – DCM, ADLB, TMB: designed the research (project conception, development of overall research plan, and study oversight); DCM, MAP: conducted the research (hands-on conduct of the experiments and data collection); DCM, MAP: provided essential databases necessary for the research; DCM: analyzed data or performed statistical analysis; DCM, MAP, VKT, NAM, MFN, ADLB: wrote the article (only authors who made a major contribution); DCM: had primary responsibility for final content; DLC: provided analytical methodology expertise; DLC, NAM, MFN, JNK, TMB, KEN, ADLB: provided technical expertise; DCM, MAP, VKT, TMB, JNK, DLC, NAM, MFN, KEN, ADLB: read and approved the final manuscript.

## Data availability

Data described in the manuscript, code book, and analytic code will be made available upon request pending application and approval.

## Declaration of generative AI and AI-assisted technologies in the writing process

During the preparation of this work, no author used such tools.

## Funding

This project was partially funded by Mead Johnson Nutrition. ADLB, TMB, DLC, NAM, JNK, MFN are employees of Mead Johnson Nutrition. DCM and MAP received funding from Mead Johnson Nutrition to perform the methods and results. The employees of Mead Johnson Nutrition provided input on the study objectives, interpretation of results, and final manuscript but did not provide input on the data extraction or statistical analysis.

## Conflict of interest

DCM and MAP report financial support and writing assistance was provided by Mead Johnson Nutrition. DCM reports a relationship with Mead Johnson Nutrition that includes: consulting or advisory and funding grants. ADLB, TMB, DLC, JNC, NAM, MFN, and KEN report a relationship with Mead Johnson Nutrition that includes: employment. DCM consultants for Reckitt | Mead Johnson as an independent contractor on a separate project. The other authors declare that they have no known competing financial interests or personal relationships that could have appeared to influence the work reported in this paper. This work was sponsored by Mead Johnson Nutrition. TMB, JNK, DLC, NAM, MFN, KEN, and ADLB are all employees of this organization and contributed to the study design, interpretation of results, and manuscript writing.
