# Supplementary material for: Carbohydrate and Energy Composition of Preterm Human Milk: A Systematic Review and Meta-Analysis
Source: Adv Nutr. 2026 Aug 18;17(10):100720. doi: 10.1016/j.advnut.2026.100720 (PMC13627815; doi:10.1016/j.advnut.2026.100720)
Supplement: Supplementary file 1 [file mmc1.docx]

**Carbohydrate and Energy Composition of Preterm Human Milk: A Systematic Review and Meta-Analysis**

Corresponding Author, Ariana DL Bailey, ariana.delong13@gmail.com

**Supplementary Appendices**

**Contents**

| **Supplementary Table 1.** Data Handling Steps  **Supplementary Table 2.** Studies Reclassified Based on Analytical Methodology **Supplementary Table 3.** Sensitivity Analysis: Manuscript vs. Original Classification **Supplementary Table 4.** Meta-Analysis by Analytical Method and Milk Type **Supplementary Figure 1.** Leave-One-Out Sensitivity Analyses  **Supplementary Figure 2.** Forest Plots by Analytical Method and Milk Type  **Total Carbohydrate**  **Total Lactose**  **Energy**  **Supplementary Figure 3.** All Funnel Plots by Nutrient | **2**  **3**  **4**  **5**  **6-16**  **17-46**  **17-26**  **27-37**  **38-46**  **47-49** |
| --- | --- |

1

**Carbohydrate and Energy Composition of Preterm Human Milk: A Systematic Review and Meta-Analysis** Corresponding Author, Ariana Bailey, ariana.delong13@gmail.com

| **Supplementary Table** | **1.** | Data Handling Steps |
| --- | --- | --- |

| **Outcome** | **Statistic** | **Preferred** | **If missing** |
| --- | --- | --- | --- |
| Carbohydrate, Lactose, and Energy | Location | Mean | 1. Used median if reported |
|  |  |  | 2. Otherwise exclude |
|  | Sample Size | n (HM  samples) | 1. Used n (infants) if reported |
|  |  |  | 2. Otherwise exclude |
|  | Variability | SD | 1. Calculate SD from SEM |
|  |  |  | 2. Estimate SD from min and max |
|  |  |  | 3. Impute missing SD from available studies |
| Gestational Age | | | |
|  | Location | Mean | 1. Use median if reported |
|  |  |  | 2. Mean of min and max values |
| Age at collection | | | |
|  | Location | Mean | 1. Use median if reported |
|  |  |  | 2. Mean of min and max values |
| HM: Human Milk | | | |
| Statistic denotes the class of output summary statistics from the studies included for analysis | | | |
| For studies contributing multiple observations (e.g., measurements at multiple time points from the same mothers), estimates were aggregated to the study level using an inverse-variance weighted mean (Hedges & Olkin, 1985; DerSimonian & Laird, 1986): | | | |
|  | | | |
| Where y_i = estimate of the ith subgroup, y_i = variance of the ith subgroup | | | |
| A within-study correlation correction (ρ = 0.6) was applied to the aggregated variance to account for the non-independence of repeated measures from the same participants, following the approach described by Borenstein et al. (2009, Chapter 24): | | | |
|  | | | |
| All calculations were performed using the metafor package in R (Viechtbauer, 2010). | | | |

2

**Carbohydrate and Energy Composition of Preterm Human Milk: A Systematic Review and Meta-Analysis** Corresponding Author, Ariana Bailey, ariana.delong13@gmail.com

| **Supplementary Table 2.**  *Studies that were reclassified based on analytical methodology* | | | | | | |  |
| --- | --- | --- | --- | --- | --- | --- | --- |
| **Author** | **Year** | | | **Method** | **Original** | | **Changed To** |
| Anderson, D | 1983 | | Enzymatic | | Total Carbohydrate | | Lactose  Lactose  Lactose  Total Carbohydrate Total Carbohydrate Total Carbohydrate Total Carbohydrate Total Carbohydrate Total Carbohydrate Total Carbohydrate Total Carbohydrate Total Carbohydrate Total Carbohydrate Total Carbohydrate Total Carbohydrate |
| Perrella, S | 2015 | | Enzymatic | | Total Carbohydrate | |  |
| Sauer, C | 2017 | | LC-MS/MS | | | Total Carbohydrate |  |
| Abdulrazzaq, Y | 2003 | | Infrared | | Lactose | |  |
| Anderssen, S | 2015 | | Infrared | | Lactose | |  |
| Bulut, O | 2019 | | Infrared | | Lactose | |  |
| Groh-Wargo, S | 2016 | | Infrared | | Lactose | |  |
| Gross, S | 1983 | | Calculation | | Lactose | |  |
| Gross, S | 1987 | | Calculation | | Lactose | |  |
| Hsu, Y | 2014 | | Infrared | | Lactose | |  |
| Kreissl, A | 2016 | | Infrared | | Lactose | |  |
| Lev, H | 2014 | | Infrared | | Lactose | |  |
| Radmacher, P | 2013 | | Infrared | | Lactose | |  |
| Smilowitz, J | 2014 | | Infrared | | Lactose | |  |
| Zachariassen, G | | 2013 | Infrared | | Lactose | |  |

3

**Carbohydrate and Energy Composition of Preterm Human Milk: A Systematic Review and Meta-Analysis** Corresponding Author, Ariana Bailey, ariana.delong13@gmail.com

| **Supplementary Table 3.**  *Sensitivity analysis comparing reassigned nutrient classifications presented in the manuscript*  *against the original author-reported classifications* | | | | | | | |
| --- | --- | --- | --- | --- | --- | --- | --- |
| **Manuscript** | | | | | | **Original** | |
| **Nutrient** | **Milk Stage** | | **Estimate** | | **95% CI** | **Estimate** | **95% CI** |
| Total Carbohydrate | | All | 6.74 | | (6.41, 7.07) | 6.73 | (6.25, 7.20) |
| Total Carbohydrate | | Colostrum | 6.30 | | (6.13, 6.48) | 6.29 | (6.01, 6.56) |
| Total Carbohydrate | Transitional | | | 6.83 | (6.60, 7.06) | 6.96 | (6.53, 7.39) |
| Total Carbohydrate | | Mature | 6.80 | | (6.45, 7.14) | 6.92 | (6.40, 7.43) |
| Lactose | | All | 6.33 | | (6.02, 6.64) | 6.49 | (6.24, 6.74) |
| Lactose | | Colostrum | 5.78 | | (5.43, 6.12) | 5.84 | (5.47, 6.22) |
| Lactose | Transitional | | | 6.63 | (6.32, 6.94) | 6.66 | (6.47, 6.86) |
| Lactose | | Mature | 6.57 | | (6.26, 6.89) | 6.61 | (6.36, 6.86) |

4

**Carbohydrate and Energy Composition of Preterm Human Milk: A Systematic Review and Meta-Analysis**
Corresponding Author, Ariana Bailey, ariana.delong13@gmail.com

| **Supplementary Table 4.** Meta analysis by analytical method and milk type | | | | **Estimate** | **95% Confidence Interval** | | **95% Prediction Interval** | | **I2** | **Tau** | **p-value** |
| --- | --- | --- | --- | --- | --- | --- | --- | --- | --- | --- | --- |
| **Nutrient** | **Milk Type** | **Analytical Method** | **n** |  |  |  |  |  |  |  |  |
|  |  |  |  |  | **Lower** | **Upper** | **Lower** | **Upper** |  |  |  |

**Carbohydrate, g/100mL**

| All | Calculation | 5 | 7.32 | 7.05 | 7.58 | 6.73 | 7.90 | 80.1 | -1.00 | 0.017 |
| --- | --- | --- | --- | --- | --- | --- | --- | --- | --- | --- |
| All | Infrared | 18 | 6.50 | 6.10 | 6.90 | 4.82 | 8.18 | 99.8 | -0.01 | 1.000 |
| All | Other/Not Specified | 2 | 7.54 | 7.40 | 7.68 | 6.53 | 7.96 | 0.0 | -0.67 | 0.333 |
| Transitional | Calculation | 4 | 7.25 | 6.88 | 7.61 |  |  | 80.3 |  |  |
| Mature | Calculation | 5 | 7.29 | 7.00 | 7.57 | 6.62 | 7.96 | 88.4 | 0.40 | 0.483 |
| Colostrum | Infrared | 2 | 6.30 | 6.13 | 6.48 | 6.12 | 7.25 | 0.0 | -0.17 | 0.612 |
| Transitional | Infrared | 9 | 6.69 | 6.49 | 6.88 |  |  | 96.7 |  |  |
| Mature | Infrared | 16 | 6.55 | 6.11 | 6.98 | 4.78 | 8.31 | 99.9 | -0.18 | 0.350 |
| Mature | Other/Not Specified | 2 | 7.54 | 7.40 | 7.68 |  |  | 0.0 |  |  |

**Lactose, g/100mL**

| All | Enzymatic | 9 | 6.48 | 6.09 | 6.87 | 5.30 | 7.66 | 96.8 | 0.17 | 0.612 |
| --- | --- | --- | --- | --- | --- | --- | --- | --- | --- | --- |
| All | GC Mass Spec | 2 | 6.46 | 5.56 | 7.36 | 4.34 | 7.05 | 83.4 | 0.67 | 0.333 |
| All | HPLC | 4 | 5.70 | 5.01 | 6.38 |  |  | 81.6 |  |  |
| Colostrum | Enzymatic | 2 | 6.04 | 5.71 | 6.36 | 5.39 | 7.66 | 0.0 | -0.33 | 0.469 |
| Transitional | Enzymatic | 6 | 6.53 | 6.08 | 6.97 |  |  | 97.1 |  |  |
| Mature | Enzymatic | 7 | 6.71 | 6.35 | 7.08 | 5.74 | 7.69 | 92.2 | 0.24 | 0.562 |
| Mature | GC Mass Spec | 2 | 6.58 | 5.89 | 7.28 | 3.95 | 8.62 | 92.4 | 0.33 | 1.000 |
| Colostrum | HPLC | 2 | 5.57 | 4.89 | 6.25 |  |  | 71.2 |  |  |
| Transitional | HPLC | 2 | 6.94 | 6.49 | 7.39 |  |  | 0.0 |  |  |
| Mature | HPLC | 3 | 6.28 | 5.10 | 7.47 |  |  | 97.2 |  |  |

**Energy, kcal/100 mL**

| All | Bomb Calorimetry | 10 | 71.62 | 68.33 | 74.92 | 63.50 | 79.74 | 70.5 | -0.07 | 0.862 |
| --- | --- | --- | --- | --- | --- | --- | --- | --- | --- | --- |
| All | Calculation | 31 | 67.29 | 64.63 | 69.94 | 53.31 | 81.27 | 98.8 | 0.33 | 0.008 |
| Colostrum | Bomb Calorimetry | 3 | 63.89 | 48.68 | 79.10 | 33.97 | 93.80 | 97.1 | -0.33 | 1.000 |
| Transitional | Bomb Calorimetry | 5 | 72.45 | 67.27 | 77.63 | 60.65 | 84.25 | 95.2 | -0.20 | 0.817 |
| Mature | Bomb Calorimetry | 7 | 70.25 | 67.69 | 72.81 | 67.15 | 73.34 | 6.1 | 0.05 | 1.000 |
| Colostrum | Calculation | 5 | 63.60 | 54.91 | 72.30 | 42.57 | 84.64 | 97.6 | -0.60 | 0.233 |
| Transitional | Calculation | 19 | 68.57 | 64.56 | 72.57 | 51.24 | 85.90 | 96.9 | 0.20 | 0.233 |
| Mature | Calculation | 24 | 68.42 | 65.32 | 71.51 | 53.42 | 83.41 | 99.1 | 0.34 | 0.017 |

*Missing prediction intervals and Kendall's tau are due to small sample sizes (n < 3)*

8
